# Gender differences in the association between TyG-related index, metabolic score for insulin resistance, and overactive bladder: A cross-sectional study

**DOI:** 10.1097/MD.0000000000048109

**Published:** 2026-03-20

**Authors:** Huihua Ji, Tianbao Wang, Yi Gao

**Affiliations:** a Department of Urology, Renmin Hospital, Hubei University of Medicine, Shiyan, Hubei, P.R. China.

**Keywords:** insulin resistance, METS-IR, NHANES, OAB, TyG

## Abstract

The triglyceride-glucose (TyG) index and the metabolic score for insulin resistance (METS-IR) are emerging indicators of IR. This study aimed to explore the correlation between these emerging IR indicators and overactive bladder (OAB) in the US population. This study utilized data from a total of 12,638 participants in the 2007 to 2018 National Health and Nutrition Examination Survey. OAB symptoms were assessed using the Overactive Bladder Symptom Score, and IR was evaluated using the TyG-body mass index, TyG-waist-to-height ratio (TyG-WHtR), TyG-waist circumference, and METS-IR indices. To analyze the data, the study methods included multivariable logistic regression, subgroup analysis, restricted cubic spline curve analysis, and receiver operating characteristic curve analysis. A total of 3032 participants were diagnosed with OAB, with women accounting for approximately 67.41% of the cases. The indices TyG-body mass index, TyG-WHtR, TyG-waist circumference, and METS-IR were positively associated with OAB, with odds ratios of 1.28 (per *z*-score, 95% confidence interval [CI]: 1.22, 1.34), 1.30 (per *z*-score, 95% CI: 1.23, 1.37), 1.28 (per *z*-score, 95% CI: 1.22, 1.35), and 1.13 (per *z*-score, 95% CI: 1.07, 1.19), respectively. Furthermore, restricted cubic spline analysis indicated a nonlinear connection between TyG-WHtR and OAB, with a turning point at 3.834. Subgroup analysis further showed a stronger association among younger and middle-aged women aged 20 to 50 years. Receiver operating characteristic analysis demonstrated that TyG-WHtR had the highest area under the curve (0.642). Sensitivity analysis demonstrates the robustness of our results. This study demonstrates that the TyG-related indices and METS-IR are positively associated with OAB in the US population, particularly among younger women. Among the evaluated indices, TyG-WHtR showed the strongest diagnostic performance for OAB.

## 1. Introduction

Overactive bladder (OAB), a condition characterized by symptoms of urgency, increased urinary frequency, and nocturia, affects millions of people worldwide, severely impairing their quality of life and imposing a substantial economic burden on society.^[1,2]^ The prevalence of OAB is particularly high, especially among women.^[3]^ Recent studies suggest that neuromuscular dysfunction within the bladder and systemic metabolic disorders are both critical factors in its pathogenesis.^[4]^ Metabolic diseases, such as obesity, diabetes, and hypertension, often co-occur with OAB, indicating that shared mechanisms may exist between metabolic health and bladder dysfunction.^[5–7]^

In recent years, the understanding of metabolic diseases has significantly deepened, and insulin resistance (IR) has emerged as a key factor in systemic inflammation and metabolic disturbances.^[8]^ Traditionally associated with diseases such as type 2 diabetes and cardiovascular conditions, IR has recently been linked to bladder dysfunction as well.^[9–11]^ Among the emerging biomarkers of IR, the triglyceride-glucose (TyG) index and its variants (TyG-body mass index [TyG-BMI], TyG-waist-to-height ratio [TyG-WHtR], TyG-waist circumference [TyG-WC]), as well as the metabolic score for IR (METS-IR), have gained attention due to their simplicity, reliability, and strong correlations with metabolic syndrome (MetS) and cardiovascular outcomes. Unlike the traditional Homeostatic Model Assessment of Insulin Resistance (HOMA-IR), which requires fasting insulin measurements, the TyG index is based on readily available clinical parameters (triglycerides [TG] and fasting glucose), making it ideal for large-scale epidemiological studies. TyG-related indices incorporate measurements of body weight and fat content (such as BMI, WC, and WHtR), allowing for a more comprehensive assessment of how fat distribution modulates IR and its systemic effects.^[12]^ These indices have been proven to serve not only as predictors of cardiovascular risk but also as indicators for other metabolic diseases, such as nonalcoholic fatty liver disease and stroke, thereby highlighting their broad clinical utility.^[13,14]^ The METS-IR is a recently developed marker that reflects insulin sensitivity and its metabolic impact. By integrating lipid levels, fasting glucose, and anthropometric measures, METS-IR provides a comprehensive evaluation of an individual’s metabolic health status.^[15]^ Recent studies have shown that METS-IR is associated with an increased risk of adverse cardiovascular outcomes, MetS, and all-cause mortality.^[16]^ However, the role of METS-IR in bladder health and OAB remains underexplored, making it an intriguing area for further research.

Given the escalating global prevalence of MetS and OAB, it is imperative to identify metabolic biomarkers that can predict or elucidate OAB symptoms. This study aims to utilize data from the National Health and Nutrition Examination Survey (NHANES) conducted between 2007 and 2018 to investigate the association between markers of IR, specifically TyG-related indices and METS-IR, and OAB. By analyzing data from more than 12,000 participants, this research seeks to clarify the metabolic implications for OAB and determine which IR biomarkers exhibit the strongest correlation with OAB prevalence. The findings will serve as a foundation for early detection and management strategies for OAB in patients suffering from metabolic disorders.

## 2. Materials and methods

### 2.1. Data availability

A nutrition survey, laboratory tests, physical examinations, health questionnaires, and laboratory data are the 5 main components of the NHANES. Participants provide written informed consent, and the database is available to researchers, thereby facilitating multiple studies and informing health policy.

### 2.2. Study population

**Figure 1.**
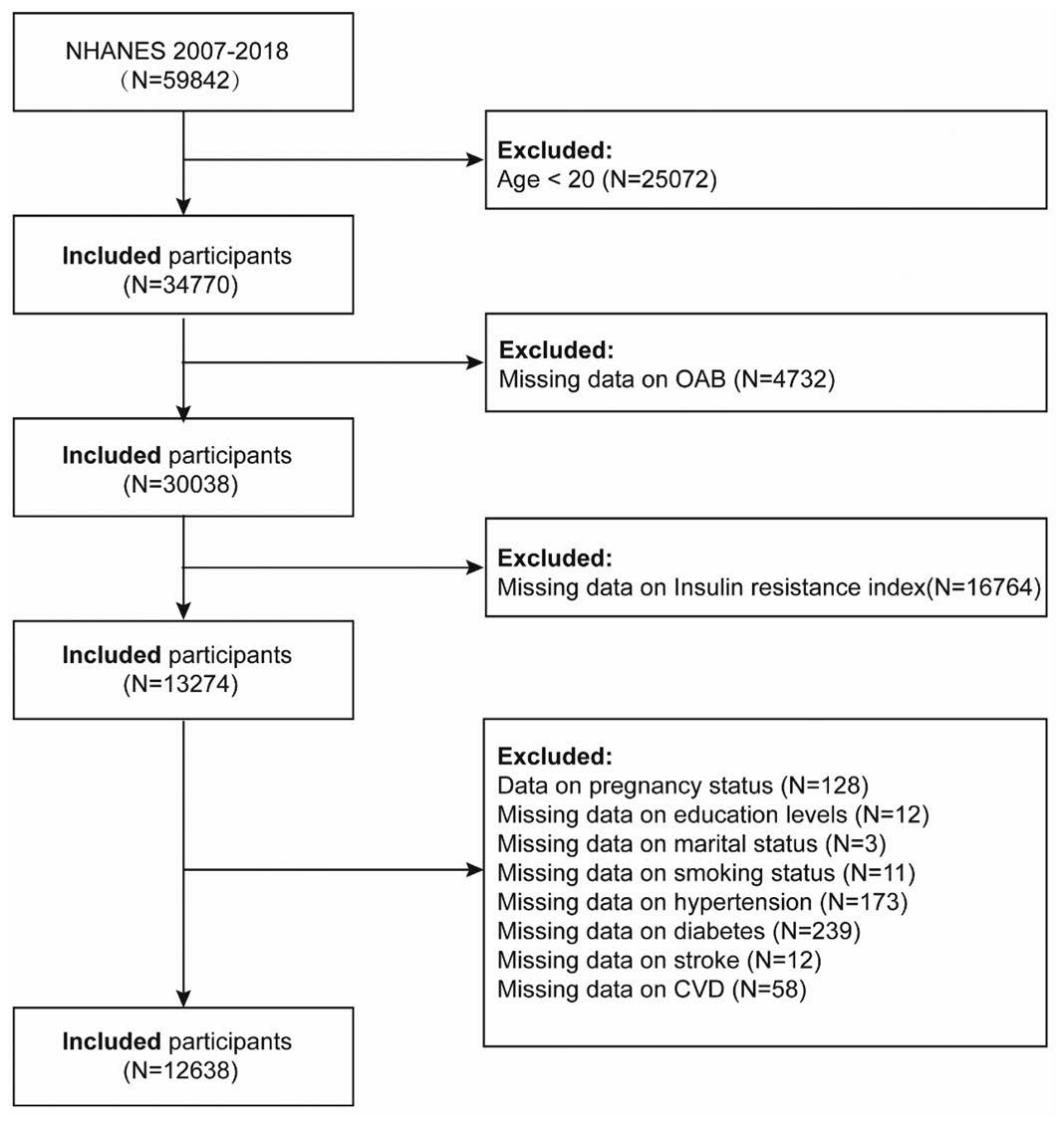
Study population flowchart 2007 to 2018. CVD = cardiovascular disease, NHANES = National Health and Nutrition Examination Survey, OAB = overactive bladder.

The study initially included 59,842 participants. The following were the exclusion criteria: individuals under the age of 20 (n = 25,072); missing OAB data (n = 4732); missing IR index data (n = 16,764); currently pregnant participants (n = 128); missing education data (n = 12); lacking information about marital status (n = 3); missing information on smoking status (n = 11); missing hypertension data (n = 173); missing diabetes data (n = 239); missing stroke data (n = 12); and missing cardiovascular disease (CVD) data (n = 58; Fig. 1).

### 2.3. Assessment of METS-IR and TyG-related indices

In the mobile examination center, trained technicians measured BMI, WC, and height. TG and high-density lipoprotein cholesterol were measured utilizing the Cobas 6000 chemical analyzer, while the Roche/Hitachi Cobas C311 chemical analyzer was used to quantify fasting blood glucose (FBG). The calculation methods for the IR-related indices are as follows^[17,18]^:


$$\begin{array}{l} TyG=\ln\left(TG\;\left[mg/dL\right]\times FBG\;\left[mg/dL\right]/2\right) \end{array}$$

$$\begin{array}{l} TyG-BMI=TyG\times BMI \end{array}$$

$$\begin{array}{l} \;TyG-WHtR=TyG\times WHtR\; \end{array}$$

$$\begin{array}{l} TyG-WC=TyG\times WC\; \end{array}$$

$$\begin{array}{l} METS-IR=\frac{\ln\left(\left[2\times FBG\;\left[mg/dL\right]+TG\;\left[mg/dL\right]\right]\times BMI\right)}{\ln\left(HDL-C\;\left[mg/dL\right]\right)} \end{array}$$

### 2.4. Evaluation of OAB

An important tool for assessing OAB symptoms is the “Kidney Conditions-Urology Questionnaire,” which is used by the NHANES database to evaluate symptoms related to the condition. Specifically, participants are asked questions such as, “Did you ever leak or lose control when you felt the need to urinate but were unable to reach a toilet promptly during the last 12 months?” and “How often did this happen?” These questions are used to assess the presence and frequency of OAB symptoms. In order to gauge how severe nocturia was, subjects were asked, “How often did you wake up throughout the night to go to the bathroom from the time you went to bed until you woke up in the morning during the last 30 days?” To determine the severity of OAB in further detail, the Overactive Bladder Symptom Score was employed. Individuals with an overall score of 3 or more in this study were considered to have OAB.^[19–21]^ The scoring criteria used to convert NHANES symptom frequencies into Overactive Bladder Symptom scores are presented in Table S1, Supplemental Digital Content, https://links.lww.com/MD/R559.

### 2.5. Covariate evaluation

In this study, covariates included gender (male/female), age, and race. Three categories were used to classify educational attainment: less than high school, high school, and more than high school. There were 3 categories for marital status: never married, married and cohabiting, and widowed, divorced, or separated. Three categories were established for the poverty-income ratio: <1.3 (poor income), 1.3 to 3.5 (middle income), and >3.5 (high income). Three categories were created based on BMI: <25 kg/m^2^, 25 to 30 kg/m^2^, and ≥30 kg/m^2^. There were 3 categories for smoking status: never (≤100 cigarettes or never smoked), former (≥100 cigarettes but no longer smoking), and current (≥100 cigarettes and actively smoking). Anyone who has drunk 12 or more alcoholic beverages in a single year of their lives was considered a drinker. A medical diagnosis of diabetes was defined as having a FBG level of 126 mg/dL or higher. A history of hypertension, current antihypertensive drug use, or an average of 3 blood pressure measurements of ≥140/90 mm Hg were all considered indicators of hypertension. Stroke was categorized as “yes” or “no.” CVD was categorized as “yes” or “no.”

### 2.6. Sensitivity analysis

We re-included the HOMA-IR index, caffeine intake, carbohydrate intake, and depression scores as covariates for sensitivity analysis. HOMA-IR calculation formula: FBG (mmol/L) × insulin (μU/mL)/22.5. Depressive symptoms and severity were assessed using the patient health questionnaire-9 scale. The patient health questionnaire-9 is a self-administered questionnaire developed based on the 9 diagnostic criteria and symptoms of depression listed in the Diagnostic and Statistical Manual of Mental Disorders, Fifth Edition. The cumulative score ranges from 0 to 27, with higher scores indicating a greater likelihood of depression. Caffeine and carbohydrate intake were obtained via 24-hour dietary recall interviews.^[22]^ During the mobile examination center assessment period, trained interviewers conducted dietary recall interviews using an automated data collection system.^[23,24]^

### 2.7. Statistical analysis

Statistical analyses followed the data analysis guidelines provided by NHANES, using appropriate NHANES complex multistage sampling weights. For continuous variables, we reported survey-weighted means (95% confidence interval [CI]), and for categorical variables, we reported survey-weighted percentages (95% CI). The OAB and non-OAB groups were compared using weighted chi-square tests or weighted linear regression. In this study, all anthropometric variables were standardized using the following *z*-score transformation: *z*-score = (index−index_mean)/index_sd.

This study employed 3 weighted logistic regression models to explore the associations between IR–related indices (TyG-BMI, TyG-WHtR, TyG-WC, and METS-IR) and OAB. Model 1 was unadjusted, model 2 was adjusted for demographic variables (age, sex, and race/ethnicity), and model 3 further included socioeconomic, lifestyle, and clinical factors (educational level, marital status, BMI, poverty-income ratio, smoking, alcohol consumption, diabetes, hypertension, CVD, and stroke). After adjustment, restricted cubic spline (RCS) analysis was applied to assess the potential nonlinear relationships between these indices and OAB. Subgroup analyses and interaction tests were performed using the covariates listed in the baseline table to evaluate heterogeneity across subgroups. Finally, the predictive performance of METS-IR and TyG-related indices for OAB was assessed by receiver operating characteristic (ROC) curve analysis, and their area under the curve (AUC) values were compared. All statistical analyses were conducted using R version 4.3.3 (R Foundation for Statistical Computing) and EmpowerStats version 4.20 (X&Y Solutions, Inc.), with statistical significance defined as *P* < .05.

## 3. Results

### 3.1. Baseline characteristics of the study population

The study included 12,638 participants in total, with a weighted prevalence of OAB of 20.21% (95% CI: 19.18%, 21.28%). Participants with OAB demonstrated significantly higher *z*-scores for TyG-BMI, TyG-WHtR, TyG-WC, and METS-IR compared with those without OAB, with values of 0.26, 0.34, 0.28, and 0.06, respectively (*P* < .01). Moreover, the OAB group had a higher proportion of women, individuals with a high school education, those who were widowed/divorced/separated, those with a BMI ≥ 30, smokers, and individuals with hypertension, diabetes, or a history of stroke (Table 1).

**Table 1:** Weighted baseline characteristics of the study population.

Characteristics	Total (n = 12,638)	Non-OAB (n = 9606)	OAB (n = 3032)	*P*-value
Age (yr)	48.03 (47.49, 48.56)	45.59 (45.03, 46.16)	57.63 (56.86, 58.40)	<.0001
Gender (%)				<.0001
Male	50.02 (48.98, 51.07)	54.44 (53.19, 55.69)	32.59 (30.25, 35.02)	
Female	49.98 (48.93, 51.02)	45.56 (44.31, 46.81)	67.41 (64.98, 69.75)	
Race (%)				<.0001
Mexican American	8.57 (7.18, 10.20)	8.89 (7.43, 10.60)	7.32 (5.99, 8.92)	
Other Hispanic	5.84 (4.85, 7.02)	6.07 (4.98, 7.37)	4.94 (4.07, 5.98)	
Non-Hispanic White	68.23 (65.47, 70.88)	68.00 (65.24, 70.65)	69.14 (65.60, 72.47)	
Non-Hispanic Black	10.05 (8.74, 11.53)	9.27 (8.06, 10.63)	13.13 (11.11, 15.46)	
Other Race	7.31 (6.48, 8.23)	7.77 (6.85, 8.80)	5.47 (4.40, 6.78)	
Education level (%)				<.0001
<High school	15.73 (14.44, 17.12)	14.12 (12.81, 15.53)	22.11 (20.29, 24.05)	
High school	23.01 (21.64, 24.44)	22.33 (20.87, 23.87)	25.70 (23.05, 28.53)	
>High school	61.25 (59.12, 63.35)	63.55 (61.27, 65.77)	52.19 (49.32, 55.04)	
Marital status (%)				<.0001
Never married	17.69 (16.43, 19.03)	19.68 (18.30, 21.13)	9.84 (8.42, 11.47)	
Married/living with partner	64.13 (62.55, 65.68)	64.64 (63.04, 66.21)	62.09 (59.27, 64.83)	
Widowed/divorced/separated	18.18 (17.18, 19.24)	15.68 (14.68, 16.74)	28.07 (25.89, 30.36)	
PIR (%)				<.0001
<1.3	21.86 (20.30, 23.49)	20.60 (19.09, 22.21)	26.80 (24.13, 29.64)	
1.3–3.5	35.28 (33.80, 36.78)	34.50 (32.88, 36.17)	38.34 (36.05, 40.69)	
≥3.5	42.86 (40.69, 45.07)	44.89 (42.74, 47.06)	34.86 (31.33, 38.56)	
BMI (%)				<.0001
<25	29.75 (28.40, 31.13)	31.86 (30.42, 33.34)	21.40 (19.29, 23.66)	
25–30	33.24 (32.20, 34.29)	34.04 (32.89, 35.22)	30.05 (27.84, 32.36)	
≥30	37.02 (35.70, 38.35)	34.09 (32.64, 35.58)	48.55 (46.35, 50.76)	
Smoking status (%)				.0022
Never	54.69 (53.10, 56.27)	55.77 (54.17, 57.36)	50.41 (47.03, 53.79)	
Now	19.37 (18.16, 20.65)	19.09 (17.83, 20.42)	20.48 (18.32, 22.83)	
Former	25.94 (24.62, 27.30)	25.14 (23.76, 26.56)	29.11 (26.33, 32.05)	
Alcohol intake (%)				<.0001
No	9.73 (8.67, 10.89)	8.83 (7.76, 10.03)	13.27 (11.40, 15.38)	
Yes	90.27 (89.11, 91.33)	91.17 (89.97, 92.24)	86.73 (84.62, 88.60)	
Hypertension (%)				<.0001
No	61.40 (59.92, 62.85)	66.42 (64.85, 67.96)	41.55 (39.10, 44.05)	
Yes	38.60 (37.15, 40.08)	33.58 (32.04, 35.15)	58.45 (55.95, 60.90)	
Diabetes (%)				<.0001
No	85.33 (84.40, 86.23)	88.50 (87.53, 89.40)	72.85 (70.46, 75.12)	
Yes	14.67 (13.77, 15.60)	11.50 (10.60, 12.47)	27.15 (24.88, 29.54)	
Stroke (%)				<.0001
No	97.15 (96.74, 97.51)	98.10 (97.75, 98.40)	93.38 (92.15, 94.43)	
Yes	2.85 (2.49, 3.26)	1.90 (1.60, 2.25)	6.62 (5.57, 7.85)	
CVD (%)				<.0001
No	92.85 (92.18, 93.47)	94.89 (94.25, 95.46)	84.81 (82.94, 86.51)	
Yes	7.15 (6.53, 7.82)	5.11 (4.54, 5.75)	15.19 (13.49, 17.06)	
Height (cm)	168.95 (168.69, 169.20)	169.87 (169.59, 170.14)	165.32 (164.78, 165.87)	<.0001
WC (cm)	99.50 (98.97, 100.03)	98.24 (97.69, 98.78)	104.48 (103.63, 105.34)	<.0001
WHtR	0.59 (0.59, 0.59)	0.58 (0.58, 0.58)	0.63 (0.63, 0.64)	<.0001
FBG (mg/dL)	107.45 (106.65, 108.25)	105.50 (104.72, 106.29)	115.15 (113.36, 116.94)	<.0001
HDL-C (mg/dL)	54.09 (53.55, 54.63)	53.92 (53.32, 54.52)	54.77 (53.89, 55.64)	.0889
TG (mg/dL)	124.34 (121.54, 127.15)	122.56 (119.52, 125.61)	131.37 (126.76, 135.98)	.0006
TyG	8.57 (8.56, 8.59)	8.55 (8.53, 8.57)	8.68 (8.65, 8.71)	<.0001
TyG-BMI	247.87 (246.03, 249.71)	243.44 (241.45, 245.43)	265.36 (261.96, 268.75)	<.0001
TyG-WHtR	5.07 (5.04, 5.10)	4.97 (4.93, 5.00)	5.48 (5.42, 5.54)	<.0001
TyG-WC	854.72 (849.33, 860.10)	841.86 (836.17, 847.55)	905.48 (896.50, 914.45)	<.0001
METS-IR	2.32 (2.31, 2.33)	2.31 (2.31, 2.32)	2.34 (2.33, 2.35)	.0001
TyG-BMI *z*-score	−0.03 (−0.06, 0.01)	−0.10 (−0.13, −0.06)	0.26 (0.20, 0.32)	<.0001
TyG-WHtR *z*-score	−0.06 (−0.10, −0.03)	−0.17 (−0.20, −0.13)	0.34 (0.28, 0.39)	<.0001
TyG-WC *z*-score	−0.02 (−0.05, 0.01)	−0.09 (−0.13, −0.06)	0.28 (0.23, 0.33)	<.0001
METS-IR *z*-score	−0.04 (−0.07, −0.00)	−0.06 (−0.10, −0.03)	0.06 (0.01, 0.11)	.0001

For continuous variables, survey-weighted means (95% CI) were reported, and *P*-values were calculated using survey-weighted linear regression (svyglm). For categorical variables, survey-weighted percentages (95% CI) were reported, and *P*-values were calculated using the survey-weighted Chi-square test (svytable).

BMI = body mass index, CI = confidence interval, CVD = cardiovascular disease, FBG = fasting blood glucose, HDL-C = high-density lipoprotein cholesterol, METS-IR = metabolic score for insulin resistance, OAB = overactive bladder, PIR = poverty-income ratio, TG = triglyceride, TyG = triglyceride-glucose, TyG-BMI = triglyceride-glucose body mass index, TyG-WC = triglyceride-glucose waist circumference, TyG-WHtR = triglyceride-glucose waist-to-height ratio, WC = waist circumference, WHtR = waist-to-height ratio.

### 3.2. The relationship between TyG-related indices, METS-IR, and OAB

**Figure 2.**
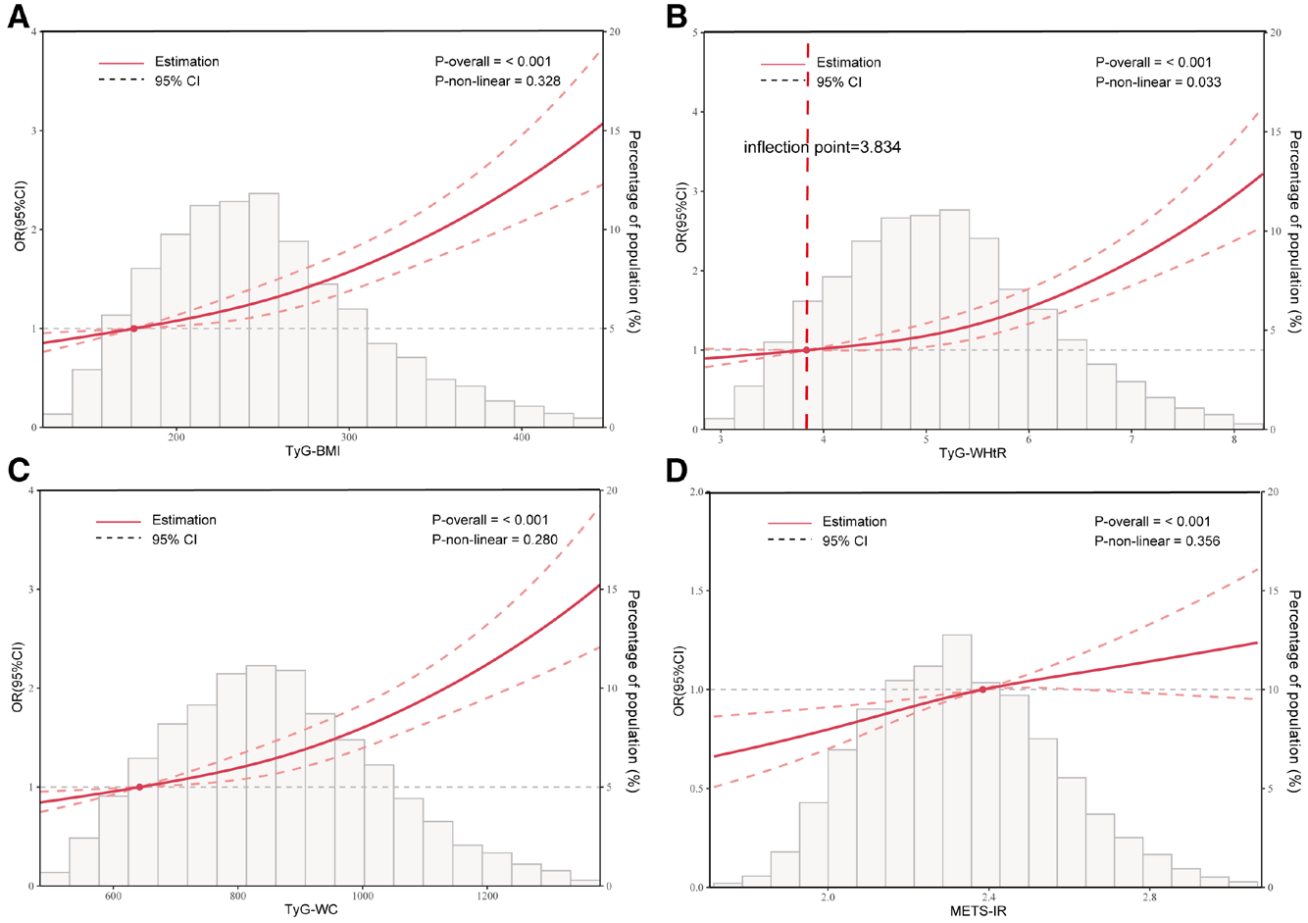
Dose-response relationship between TyG-related indices, METS-IR, and OAB. Age, gender, race, education level, marital status, PIR, smoking status, alcohol consumption, diabetes, hypertension, CVD, and stroke were adjusted. (A) TyG-BMI and OAB; (B) TyG-WHtR and OAB; (C) TyG-WC and OAB; and (D) METS-IR and OAB. CI = confidence interval, CVD = cardiovascular disease, METS-IR = metabolic score for insulin resistance, OAB = overactive bladder, OR = odds ratio, PIR = poverty-income ratio, TyG = triglyceride-glucose, TyG-BMI = triglyceride-glucose body mass index, TyG-WC = triglyceride-glucose waist circumference, TyG-WHtR = triglyceride-glucose waist-to-height ratio.

A positive association was found between METS-IR, TyG-related indicators, and OAB, according to the results of the multivariate logistic regression analysis. In model 3, for every 1 *z*-score increase in TyG-BMI, the likelihood of OAB increased by 28% (odds ratio [OR] = 1.28, 95% CI: 1.22, 1.34). Similarly, for each 1 *z*-score increase in TyG-WHtR, the likelihood of OAB increased by 30% (OR = 1.30, 95% CI: 1.23, 1.37), and for each 1 *z*-score increase in TyG-WC, the OR for OAB was 1.28 (95% CI: 1.22, 1.35). For each 1 *z*-score increase in METS-IR, the OR for OAB was 1.13 (95% CI: 1.07, 1.19). Moreover, compared to individuals in the lowest quartile (Q1), those in the highest quartile had a considerably higher likelihood of having OAB (*P* for trend < .05; Table 2). Additionally, RCS analysis revealed that there was no nonlinear correlation between TyG-BMI, TyG-WC, and METS-IR with OAB (nonlinear *P*-value > .05). However, an L-shaped nonlinear connection was found between TyG-WHtR and OAB, with a critical value of 3.834 (nonlinear *P*-value = .033; Fig. 2, Table 3).

**Table 2:** Weighted multivariable logistic regression analysis of the association between TyG-related indices, METS-IR, and OAB.

Characteristic	Model 1		Model 2		Model 3	
	OR (95% CI)	*P*-value	OR (95% CI)	*P*-value	OR (95% CI)	*P*-value
TyG-BMI *z*-score						
Continuous	1.42 (1.36, 1.48)	<.001	1.39 (1.33, 1.45)	<.001	1.28 (1.22, 1.34)	<.001
Q1	Reference		Reference		Reference	
Q2	1.44 (1.27, 1.63)	<.001	1.22 (1.06, 1.40)	.005	1.19 (1.03, 1.36)	.017
Q3	1.77 (1.57, 2.01)	<.001	1.50 (1.31, 1.72)	<.001	1.35 (1.17, 1.55)	<.001
Q4	2.60 (2.31, 2.94)	<.001	2.28 (2.00, 2.60)	<.001	1.84 (1.60, 2.12)	<.001
*P* for trend		<.001		<.001		<.001
TyG-WHtR *z*-score						
Continuous	1.66 (1.59, 1.73)	<.001	1.45 (1.38, 1.52)	<.001	1.30 (1.23, 1.37)	<.001
Q1	Reference		Reference		Reference	
Q2	1.70 (1.48, 1.94)	<.001	1.21 (1.05, 1.40)	.011	1.16 (1.00, 1.35)	.043
Q3	2.32 (2.04, 2.65)	<.001	1.54 (1.34, 1.78)	<.001	1.35 (1.16, 1.56)	<.001
Q4	3.88 (3.42, 4.41)	<.001	2.41 (2.10, 2.76)	<.001	1.83 (1.58, 2.12)	<.001
*P* for trend		<.001		<.001		<.001
TyG-WC *z*-score						
Continuous	1.47 (1.42, 1.54)	<.001	1.42 (1.36, 1.49)	<.001	1.28 (1.22, 1.35)	<.001
Q1	Reference		Reference		Reference	
Q2	1.50 (1.32, 1.71)	<.001	1.19 (1.03, 1.37)	.017	1.12 (0.97, 1.29)	.122
Q3	2.14 (1.88, 2.42)	<.001	1.68 (1.47, 1.93)	<.001	1.46 (1.27, 1.68)	<.001
Q4	2.86 (2.53, 3.23)	<.001	2.34 (2.05, 2.68)	<.001	1.79 (1.55, 2.07)	<.001
*P* for trend		<.001		<.001		<.001
METS-IR *z*-score						
Continuous	1.13 (1.08, 1.17)	<.001	1.29 (1.24, 1.36)	<.001	1.13 (1.07, 1.19)	<.001
Q1	Reference		Reference		Reference	
Q2	1.20 (1.07, 1.35)	.003	1.32 (1.16, 1.51)	<.001	1.23 (1.08, 1.40)	.002
Q3	1.27 (1.12, 1.42)	<.001	1.56 (1.37, 1.77)	<.001	1.27 (1.11, 1.46)	<.001
Q4	1.41 (1.25, 1.58)	<.001	1.98 (1.74, 2.26)	<.001	1.39 (1.21, 1.60)	<.001
*P* for trend		<.001		<.001		<.001

Model 1: no covariates were adjusted.

Model 2: adjusted for age, gender, and race.

Model 3: adjusted for age, gender, race, education level, marital status, PIR, smoking status, alcohol consumption, diabetes, hypertension, CVD, and stroke.

CI = confidence interval, CVD = cardiovascular disease, METS-IR = metabolic score for insulin resistance, OAB = overactive bladder, OR = odds ratio, PIR = poverty-income ratio, TyG = triglyceride-glucose, TyG-BMI = triglyceride-glucose body mass index, TyG-WC = triglyceride-glucose waist circumference, TyG-WHtR = triglyceride-glucose waist-to-height ratio.

**Table 3:** Threshold effect analysis of TYG-WHtR and OAB.

TYG-WHtR	Adjusted OR (95% CI)	*P*-value
Inflection point	3.834	
<3.834	1.20 (1.09, 1.33)	.001
>3.834	1.45 (1.32, 1.59)	<.001
Log likelihood ratio test		.021

Age, gender, race, education level, marital status, PIR, smoking status, alcohol consumption, diabetes, hypertension, CVD, and stroke were adjusted.

CI = confidence interval, CVD = cardiovascular disease, OAB = overactive bladder, OR = odds ratio, PIR = poverty-income ratio, TyG-WHtR = triglyceride-glucose waist-to-height ratio.

### 3.3. Subgroup analysis

**Figure 3.**
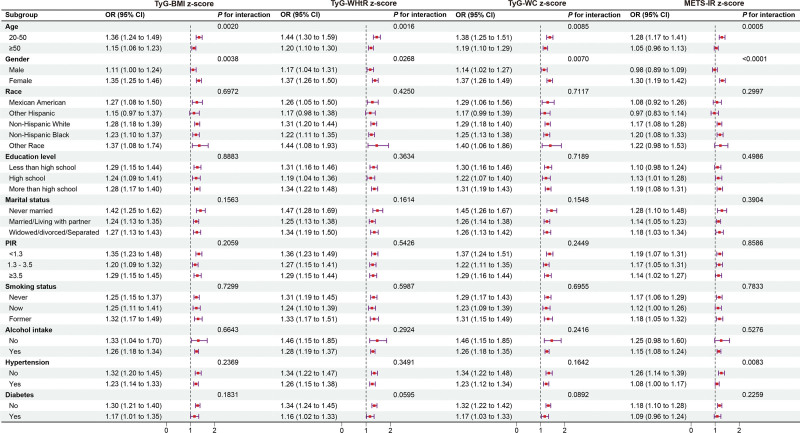
Subgroup analysis of TyG-related index, METS-IR, and OAB. The above model was adjusted for gender, age, race, education level, marital status, PIR, smoking status, alcohol consumption, diabetes, hypertension, cardiovascular disease (CVD), and stroke. For each analysis, the model was not adjusted for the corresponding stratification variable. CI = confidence interval, METS-IR = metabolic score for insulin resistance, OAB = overactive bladder, OR = odds ratio, PIR = poverty-income ratio, TyG = triglyceride-glucose, TyG-BMI = triglyceride-glucose body mass index, TyG-WC = triglyceride-glucose waist circumference, TyG-WHtR = triglyceride-glucose waist-to-height ratio.

The findings showed that, for all groupings, there was a positive correlation between METS-IR, OAB prevalence, and TyG-related indicators. Notably, the positive association between TyG-BMI and OAB prevalence was stronger among individuals aged 20 to 50 years (per *z*-score, OR = 1.36, 95% CI: 1.24, 1.49) and women (per *z*-score, OR = 1.35, 95% CI: 1.25, 1.46). Similarly, the favorable correlation between TyG-WHtR and OAB prevalence was stronger among individuals aged 20 to 50 years (per *z*-score, OR = 1.44, 95% CI: 1.30, 1.59) and women (per *z*-score, OR = 1.37, 95% CI: 1.26, 1.50). Likewise, the correlation between TyG-WC and OAB prevalence was stronger among individuals aged 20 to 50 years (per *z*-score, OR = 1.38, 95% CI: 1.25, 1.51) and women (per *z*-score, OR = 1.37, 95% CI: 1.26, 1.49). Moreover, the positive connection between METS-IR and OAB prevalence was also stronger among individuals aged 20 to 50 years (per *z*-score, OR = 1.28, 95% CI: 1.17, 1.41) and women (per *z*-score, OR = 1.30, 95% CI: 1.19, 1.42; *P* for interaction < .05; Fig. 3).

### 3.4. ROC analysis

**Figure 4.**
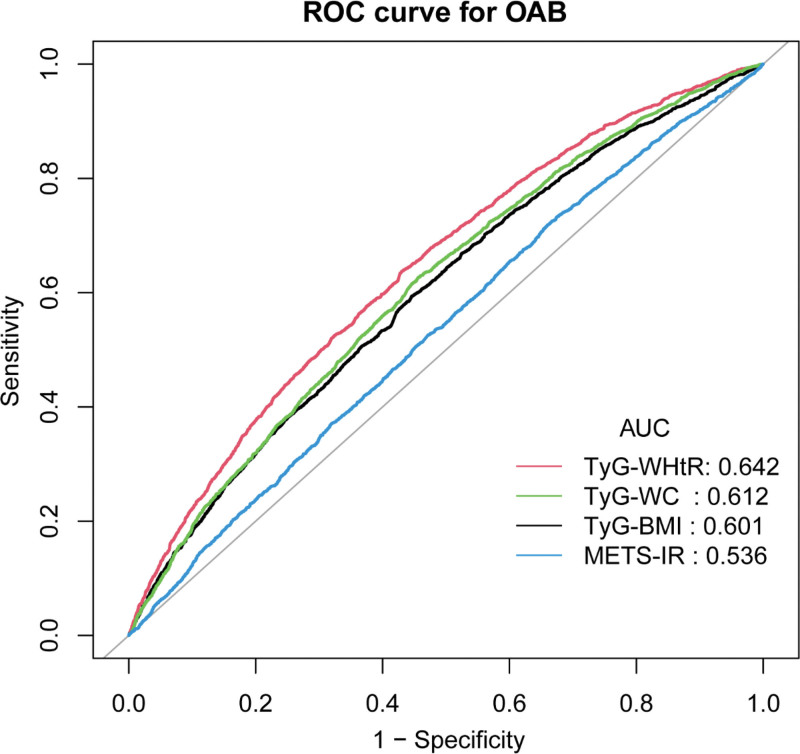
ROC curves and the AUC values of insulin resistance-related indices (TyG-BMI, TyG-WHtR, TyG-WC, METS-IR) in diagnosing OAB. AUC = area under the curve, OAB = overactive bladder, ROC = receiver operating characteristic, TyG-BMI = triglyceride-glucose body mass index, TyG-WC = triglyceride-glucose waist circumference, TyG-WHtR = triglyceride-glucose waist-to-height ratio.

The diagnostic performance of METS-IR and TyG-related indicators in identifying OAB patients was assessed in this study using ROC curves. The analysis revealed that TyG-WHtR demonstrated significantly higher diagnostic ability for OAB compared with TyG-BMI, TyG-WC, and METS-IR. The AUC for TyG-BMI, TyG-WHtR, TyG-WC, and METS-IR were 0.601, 0.642, 0.612, and 0.536, respectively. These results suggest that TyG-WHtR possesses greater discriminative ability and accuracy in predicting OAB risk compared with the other IR indices (TyG-BMI, TyG-WC, and METS-IR; Fig. 4, Table 4).

**Table 4:** Comparison of AUC values for TyG-related indices and METS-IR.

Test	AUC	95% CI low	95% CI upp	Best threshold	Specificity	Sensitivity	*P* for different in AUC
TyG-BMI	0.6010	0.5895	0.6125	245.3624	0.5538	0.5943	Reference
TyG-WHtR	0.6423	0.6312	0.6535	5.1296	0.5697	0.6375	<.001
TyG-WC	0.6117	0.6004	0.6230	849.8157	0.5438	0.6266	<.001
METS-IR	0.5363	0.5245	0.5480	2.2095	0.3400	0.7187	<.001

AUC = area under the curve, CI = confidence interval, METS-IR = metabolic score for insulin resistance, TyG-BMI = triglyceride-glucose body mass index, TyG-WC = triglyceride-glucose waist circumference, TyG-WHtR = triglyceride-glucose waist-to-height ratio.

### 3.5. Sensitivity analysis

Sensitivity analysis revealed that IR-related indicators remained positively correlated with OAB even after incorporating relevant covariates (Tables S2 and S3, Supplemental Digital Content, https://links.lww.com/MD/R559). ROC analysis indicates that compared with other IR indicators (TyG-BMI, TyG-WC, METS-IR, and HOMA-IR), TyG-WHtR demonstrates superior discriminatory ability and accuracy in predicting OAB risk, confirming the robustness of the findings (Fig. S1, Supplemental Digital Content, https://links.lww.com/MD/R559).

## 4. Discussion

This research looked into the correlation between IR-related indices and OAB in a cohort of 12,638 participants. A weighted multivariable logistic regression analysis identified a notable correlation between TyG-BMI, TyG-WHtR, TyG-WC, METS-IR, and the incidence of OAB. The adjusted results confirmed that these associations remained significant, with the strongest correlations observed in the highest quartiles of TyG-BMI, TyG-WHtR, and TyG-WC. Moreover, RCS curve analysis showed no nonlinear relationship between TyG-BMI, TyG-WC, METS-IR, and OAB (*P*-nonlinear > .05). However, a nonlinear relationship was observed between TyG-WHtR and OAB. These findings suggest that distinct mechanisms may underlie the diagnostic value of IR-related indices for OAB. ROC curves and AUC values showed that TyG-WHtR performed better as a diagnostic tool for OAB than TyG-BMI, TyG-WC, and METS-IR. Subgroup analysis revealed that age and gender may modify these associations, with a stronger correlation between IR-related indices and OAB observed in women under 50 years of age.

Prior research has examined the connection between IR and OAB, suggesting that IR plays a crucial role in inflammation, obesity, and OAB.^[25,26]^ Individuals with elevated IR often exhibit obesity, making IR a key indicator to monitor in the development of OAB. Moreover, the use of IR-related indices has garnered increasing attention. These indices have been validated for their diagnostic performance in MetS, metabolic dysfunction-related diseases, and CVDs.^[27]^ Furthermore, research has shown that MetS may be a contributing mechanism to OAB development, with MetS-related subtypes appearing in various forms of OAB.^[7,28]^ This may be related to bladder mucosal IR caused by dysregulated nutrient-sensing pathways. Additionally, TyG, as an IR assessment index, has been increasingly applied in recent years,^[29,30]^ particularly when combined with obesity indices such as TyG-BMI, TyG-WHtR, and TyG-WC.^[31]^ Compared with TyG alone, this combination’s ability to evaluate IR was noticeably better. This highlights that combining TyG-related markers with obesity indices offers a more effective reflection of the degree of IR than using TyG alone as a surrogate marker.^[32]^ Furthermore, it is feasible to gain a deeper understanding of the impact of various fat distributions on IR and associated OAB by integrating TyG with obesity markers. Two large cohort studies and Mendelian randomization have demonstrated a causal relationship between TyG and a reduced risk of stroke, suggesting that TyG plays a pivotal role in stroke risk. Furthermore, TyG has been identified as a key factor in predicting stroke occurrence.^[33]^ A prospective cohort study demonstrates that both TyG-WC and TyG-WHtR greatly increase the diagnostic accuracy and prediction of CVD mortality when compared to the already widely used TyG. These results are especially significant for improving risk stratification, lowering screening expenses, and identifying groups at early risk of CVD.^[34]^

In middle-aged and older T2D patients in the US, a prospective study found a U-shaped correlation between the TyG index and death. It is noteworthy that the TyG index becomes strongly correlated with death once the threshold is crossed, underscoring the need to preserve ideal levels of this index in clinical practice.^[35]^ According to a study, the TyG index and cardiovascular events in young and middle-aged persons living in the community are independently correlated. Moreover, the degree of obesity increases the strength of this connection. Therefore, while evaluating and treating young and middle-aged obese individuals, it is critical to take IR into account to decrease future cardiovascular events in this population.^[35]^ In the US population, there is a correlation between an elevated METS-IR index and a higher risk of 3 different forms of urinary incontinence. Interestingly, women are more likely than males to have stress urinary incontinence when their METS-IR score is higher.^[36]^ In the general population, a high METS-IR score was significantly correlated with depressed symptoms, according to a cross-sectional study. With an elevated METS-IR score, women and middle-aged and older persons are particularly more likely to display depressive symptoms.^[17]^

Earlier research has shown that TyG-BMI has been widely applied as a predictive tool for various diseases, including cancer and inflammation.^[37,38]^ In this study, RCS curve analysis revealed that TyG-BMI might be an effective means of diagnosing OAB, consistent with previous findings. Similarly, TyG-WHtR and TyG-WC, as other TyG-combined obesity indices, showed similar diagnostic performance to TyG-BMI for OAB. Notably, ROC curve analysis indicated that TyG-WHtR had the highest diagnostic efficiency among the indices. Previous studies have shown that the TyG index and IR may influence bodily functions through similar mechanisms, which could be related to the development of OAB. IR leads to increased glucose absorption in the intestines, enhanced lipolysis, and elevated fatty acids, which contribute to hyperglycemia and obesity. Reactive oxygen species are produced in greater quantities by the organism when blood glucose levels are high, triggering oxidative stress reactions.^[39]^ Current research on OAB has also suggested that its development may be related to oxidative stress.^[40]^ Moreover, obesity can initiate a series of mechanisms, including chronic inflammation, hypoxia, and oxidative stress, which are consistent with the pathophysiology of OAB. Obesity further exacerbates conditions such as hypertension, decreased glucose tolerance, dyslipidemia, and IR, which may indirectly contribute to the occurrence of OAB.

Based on existing theories, we propose that IR-related indices (TyG-BMI, TyG-WHtR, TyG-WC, METS-IR) may contribute to several pathological changes in the body, including peripheral neuropathy, chronic tissue ischemia and hypoxia, inflammatory responses, dysbiosis, and hormonal metabolism disorders. These pathological changes, in turn, may exacerbate IR. Although the pathogenesis of OAB is still not entirely clear, it is thought to be connected with inflammatory responses, hormonal dysregulation, chronic diseases, and neuropsychological factors. Given these connections, we hypothesize that IR-related indices could serve as diagnostic tools for OAB, a hypothesis that has been further validated by our study.

## 5. Strengths and limitations

This study has a variety of strengths. This research is the initial investigation to use cross-sectional analysis to investigate the relationship between IR-related indices and OAB. Our findings demonstrate that TyG-BMI, TyG-WHtR, TyG-WC, and METS-IR can serve as reliable indicators for diagnosing OAB. Furthermore, compared to conventional IR markers, the IR-related indices analyzed in this study are both easily obtainable and cost-effective, providing practical and reliable tools for clinical practice. Additionally, while there is ongoing debate regarding the diagnostic efficiency of these indices for various diseases, our investigation revealed that TyG-WHtR exhibited the highest diagnostic efficiency for OAB in the US population.

Several limitations should be acknowledged. First, the reduction from the overall NHANES sample to the analytic cohort was mainly driven by the NHANES fasting subsample design (fasting TG and fasting plasma glucose are required to construct the exposure indices) and the availability of urinary symptom questionnaire modules. Although we applied the appropriate fasting subsample survey weights and accounted for the complex sampling design, selection bias may still exist, and generalizability may be limited. Second, TyG-related indices and METS-IR are surrogate markers of IR rather than gold-standard direct measurements such as the hyperinsulinemic–euglycemic clamp; moreover, composite indices incorporate adiposity and fat distribution components, and thus their metabolic interpretation should be made with caution. Third, OAB was defined using self-reported questionnaire data and symptom score mapping, which may be subject to recall bias and outcome misclassification, and NHANES lacks urodynamic testing and clinically confirmed diagnoses. Fourth, despite adjustment for multiple sociodemographic factors and major comorbidities, several important confounders could not be uniformly controlled due to database limitations, including fluid intake, use of diuretics or anticholinergic medications, history of urinary tract infection, parity and childbirth-related trauma, menopausal status and pelvic floor function, and sleep quality; therefore, residual confounding is unavoidable. Fifth, the cross-sectional design precludes causal inference and allows for reverse causation. Finally, subgroup findings are exploratory and should be interpreted cautiously given multiple comparisons; prospective and mechanistic studies are needed for confirmation.

## 6. Conclusion

TyG-BMI, TyG-WHtR, TyG-WC, and METS-IR are positively associated with the prevalence of OAB in the people of America, particularly among younger women aged 20 to 50 years. TyG-WHtR showed the best predictive value for OAB among these indices, underscoring its potential as a clinical marker for identifying high-risk populations. Future research should aim to elucidate the mechanisms by which IR contributes to OAB development and explore the potential of metabolic interventions as preventive or therapeutic strategies for OAB management.

## Acknowledgments

We would like to thank all participants in this study.

## Author contributions

**Conceptualization:** Huihua Ji, Tianbao Wang, Yi Gao.

**Methodology:** Tianbao Wang.

**Data curation:** Huihua Ji, Tianbao Wang.

**Validation:** Huihua Ji.

**Visualization:** Huihua Ji.

**Investigation:** Tianbao Wang.

**Resources:** Yi Gao.

**Supervision:** Yi Gao.

**Writing – original draft:** Huihua Ji, Tianbao Wang, Yi Gao.

**Writing – review & editing:** Huihua Ji, Yi Gao.

## Supplementary Material


