# Supplementary material for: Gender differences in the association between TyG-related index, metabolic score for insulin resistance, and overactive bladder: A cross-sectional study
Source: Medicine (Baltimore). 2026 Mar 20;105(12):e48109. doi: 10.1097/MD.0000000000048109 (PMC13008216; doi:10.1097/MD.0000000000048109)
Supplement: Supplementary file 1 [file medi-105-e48109-s001.docx]

**Supplementary Figure 1.** ROC curves and the AUC values of insulin resistance-related indices (TyG-BMI, TyG-WHtR, TyG-WC, METS-IR，HOMA-IR) in diagnosing OAB.


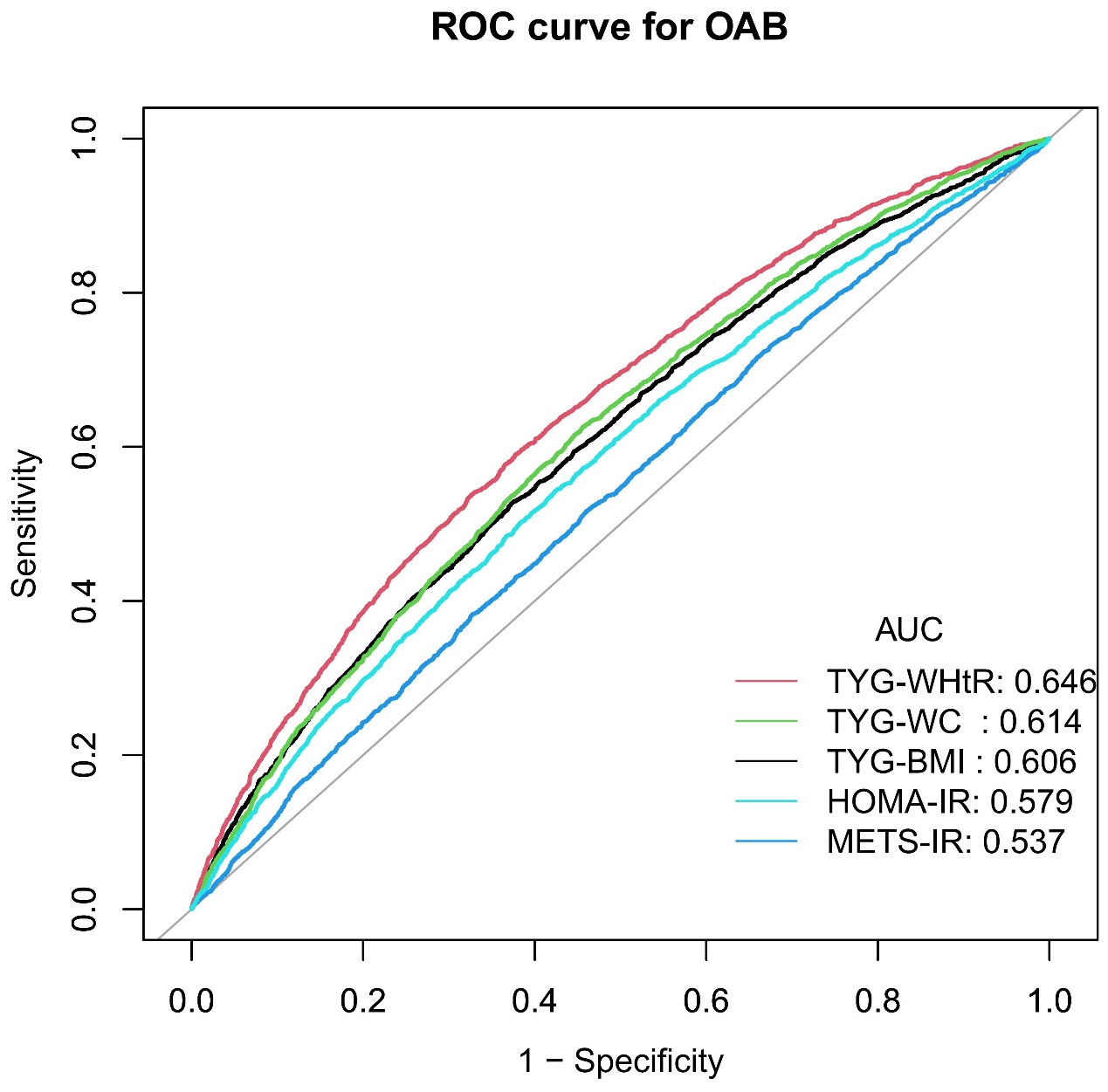


**Supplementary Table1. Criteria for Conversion of Symptom Frequencies Recorded in NHANES and OABSS Scores**

| **According to NHANES Score** | **According to OABSS Score** |
| --- | --- |
| Urge urinary incontinence frequency | Urge urinary incontinence score |
| Never | 0 |
| Less than once a month | 1 |
| A few times a month | 1 |
| A few times a week | 2 |
| Every day or night | 3 |
| Nocturia frequency | Nocturia score |
| 0 | 0 |
| 1 | 1 |
| 2 | 2 |
| 3 | 3 |
| 4 | 3 |
| 5 or more | 3 |
| When total score ≥3, the diagnosis is OAB | |

NHANES = National Health and Nutrition Examination Survey; OABSS = Overactive Bladder Symptom Score;

**Supplementary Table 2.** Baseline Population Table for Sensitivity Analysis After Adding Covariates

| **Characteristics** | **Total (n = 11456)** | **Non-OAB (n = 8961)** | **OAB (n = 2495)** | ***P*-value** |
| --- | --- | --- | --- | --- |
| Age (years) | 48.00 (47.47,48.54) | 45.57 (45.01,46.13) | 57.64 (56.86,58.42) | <0.0001 |
| Gender (%) |  |  |  | <0.0001 |
| Male | 50.13 (49.11,51.16) | 54.51 (53.28,55.72) | 32.80 (30.45,35.25) |  |
| Female | 49.87 (48.84,50.89) | 45.49 (44.28,46.72) | 67.20 (64.75,69.55) |  |
| Race (%) |  |  |  | <0.0001 |
| Mexican American | 8.59 (7.19,10.23) | 8.88 (7.42,10.61) | 7.42 (6.07,9.05) |  |
| Other Hispanic | 5.83 (4.84,7.00) | 6.07 (4.99,7.36) | 4.89 (4.03,5.91) |  |
| Non-Hispanic White | 68.31 (65.54,70.95) | 68.08 (65.31,70.73) | 69.19 (65.66,72.51) |  |
| Non-Hispanic Black | 9.97 (8.66,11.46) | 9.21 (7.99,10.58) | 12.99 (10.97,15.33) |  |
| Other Race | 7.30 (6.48,8.23) | 7.76 (6.84,8.79) | 5.50 (4.42,6.83) |  |
| Education level (%) |  |  |  | <0.0001 |
| Less than high school | 15.74 (14.44,17.12) | 14.09 (12.79,15.51) | 22.25 (20.42,24.19) |  |
| High school | 23.04 (21.65,24.49) | 22.40 (20.91,23.97) | 25.56 (22.90,28.42) |  |
| More than high school | 61.22 (59.08,63.32) | 63.50 (61.21,65.74) | 52.19 (49.29,55.08) |  |
| Marital status (%) |  |  |  | <0.0001 |
| Never married | 17.69 (16.41,19.04) | 19.67 (18.28,21.15) | 9.81 (8.39,11.44) |  |
| Married/Living with partner | 64.11 (62.52,65.68) | 64.62 (63.00,66.20) | 62.12 (59.25,64.91) |  |
| Widowed/divorced/Separated | 18.20 (17.18 ,19.26) | 15.71 (14.70 ,16.77) | 28.07 (25.83 ,30.42) |  |
| PIR (%) |  |  |  | <0.0001 |
| <1.3 | 21.80 (20.26 ,23.42) | 20.54 (19.03 ,22.15) | 26.79 (24.15 ,29.61) |  |
| 1.3 - 3.5 | 35.28 (33.83,36.76) | 34.54 (32.92,36.19) | 38.22 (35.91,40.57) |  |
| ≥3.5 | 42.92 (40.77,45.10) | 44.92 (42.78,47.08) | 34.99 (31.44,38.71) |  |
| BMI (%) |  |  |  | <0.0001 |
| <25 | 29.74 (28.38,31.13) | 31.86 (30.40,33.34) | 21.35 (19.25,23.62) |  |
| 25 - 30 | 33.32 (32.28,34.37) | 34.14 (32.97,35.32) | 30.07 (27.88,32.35) |  |
| ≥30 | 36.94 (35.62,38.29) | 34.01 (32.54,35.51) | 48.58 (46.39,50.77) |  |
| Smoking status (%) |  |  |  | 0.0029 |
| Never | 54.69 (53.11,56.27) | 55.75 (54.14,57.36) | 50.48 (47.06,53.90) |  |
| Now | 19.36 (18.15,20.64) | 19.12 (17.87,20.45) | 20.32 (18.13,22.69) |  |
| Former | 25.94 (24.64,27.29) | 25.12 (23.75,26.54) | 29.20 (26.41,32.15) |  |
| Alcohol intake (%) |  |  |  | <0.0001 |
| No | 9.77 (8.69,10.96) | 8.86 (7.77,10.08) | 13.38 (11.51,15.49) |  |
| Yes | 90.23 (89.04,91.31) | 91.14 (89.92,92.23) | 86.62 (84.51,88.49) |  |
| Hypertension (%) |  |  |  | <0.0001 |
| No | 61.46 (60.00,62.89) | 66.46 (64.91,67.98) | 41.63 (39.15,44.15) |  |
| Yes | 38.54 (37.11,40.00) | 33.54 (32.02,35.09) | 58.37 (55.85,60.85) |  |
| Diabetes (%) |  |  |  | <0.0001 |
| No | 85.39 (84.44,86.29) | 88.57 (87.59,89.47) | 72.79 (70.39,75.06) |  |
| Yes | 14.61 (13.71,15.56) | 11.43 (10.53,12.41) | 27.21 (24.94,29.61) |  |
| Stroke (%) |  |  |  | <0.0001 |
| No | 97.14 (96.73,97.50) | 98.11 (97.75,98.41) | 93.31 (92.06,94.37) |  |
| Yes | 2.86 (2.50,3.27) | 1.89 (1.59,2.25) | 6.69 (5.63,7.94) |  |
| CVD (%) |  |  |  | <0.0001 |
| No | 92.86 (92.20,93.46) | 94.90 (94.28,95.46) | 84.76 (82.89,86.47) |  |
| Yes | 7.14 (6.54,7.80) | 5.10 (4.54,5.72) | 15.24 (13.53,17.11) |  |
| Height (cm) | 168.96 (168.71,169.22) | 169.88 (169.61,170.15) | 165.33 (164.77,165.88) | <0.0001 |
| WC (cm) | 83.01 (82.41,83.62) | 82.52 (81.85,83.20) | 84.95 (83.91,85.99) | <0.0001 |
| WHtR | 0.59 (0.59,0.59) | 0.58 (0.58,0.58) | 0.63 (0.63,0.64) | <0.0001 |
| FBG(mg/dl) | 107.48 (106.66,108.30) | 105.55 (104.74,106.35) | 115.16 (113.37,116.96) | <0.0001 |
| FBG(mmol/L) | 5.97 (5.92,6.01) | 5.86 (5.81,5.90) | 6.39 (6.29,6.49) | <0.0001 |
| HDL-C(mg/dl) | 54.08 (53.54,54.61) | 53.91 (53.32,54.50) | 54.74 (53.87,55.62) | 0.088 |
| TG(mg/dl) | 124.14 (121.55,126.73) | 122.24 (119.55,124.93) | 131.68 (127.01,136.36) | 0.0001 |
| Insulin((μU/ml) | 13.18 (12.81,13.56) | 12.73 (12.31,13.15) | 14.98 (14.29,15.68) | <0.0001 |
| Depression score | 2.98 (2.87,3.09) | 2.52 (2.41,2.63) | 4.81 (4.56,5.05) | <0.0001 |
| Caffeine intake(mg/day) | 145.00 (62.00, 277.50) | 132.00 (119.00,144.50) | 170.00 (136.50,208.00) | 0.0001 |
| Carbohydrate intake(g) | 19.56(14.86, 18.26) | 16.56(14.86, 18.26) | 22.65(20.70, 24.60) | 0.005 |
|  |  |  |  |  |
| TyG | 8.59 (8.57,8.61) | 8.56 (8.54,8.58) | 8.71 (8.68,8.75) | <0.0001 |
| TyG-BMI | 250.49 (248.31,252.68) | 245.19 (242.97,247.41) | 271.49 (267.52,275.47) | <0.0001 |
| TyG-WHtR | 5.09 (5.06,5.13) | 4.98 (4.94,5.02) | 5.54 (5.47,5.60) | <0.0001 |
| TyG-WC | 859.13 (853.20,865.07) | 845.26 (839.18,851.34) | 914.12 (904.22,924.02) | <0.0001 |
| METS-IR | 2.33 (2.32,2.33) | 2.32 (2.31,2.33) | 2.35 (2.34,2.37) | <0.0001 |
| HOMA-IR | 3.81 (3.67,3.95) | 3.60 (3.44,3.76) | 4.64 (4.35,4.93) | <0.0001 |
| TyG-BMI z-score | -0.02 (-0.06,0.01) | -0.10 (-0.14,-0.07) | 0.29 (0.23,0.35) | <0.0001 |
| TyG-WHtR z-score | -0.06 (-0.10,-0.03) | -0.17 (-0.20,-0.13) | 0.36 (0.30,0.42) | <0.0001 |
| TyG-WC z-score | -0.01 (-0.05,0.02) | -0.09 (-0.13,-0.06) | 0.30 (0.24,0.35) | <0.0001 |
| METS-IR z-score | -0.04 (-0.07,-0.01) | -0.06 (-0.10,-0.03) | 0.06 (0.01,0.11) | <0.0001 |

Abbreviations: PIR, poverty-income ratio; BMI, body mass index; CVD, cardiovascular disease; WC, waist circumference; WHtR, waist-to-height ratio; HDL-C, high-density lipoprotein cholesterol; TG, triglyceride; FBG, fasting blood glucose;

OAB, overactive bladder; TyG, triglyceride-glucose index; TyG-BMI, triglyceride-glucose body mass index; TyG-WHtR, triglyceride-glucose waist-to-height ratio; TyG-WC, triglyceride-glucose waist circumference; METS-IR, metabolic score for insulin resistance. HOMA-IR，insulin resistance homeostasis model assessment.

**Supplementary Table 3.** Multiple Regression Analysis After Adding Covariates

| **Characteristic** | **Model 1** | | **Model 2** | | **Model 3** | |
| --- | --- | --- | --- | --- | --- | --- |
|  | **OR (95% CI)** | ***P* value** | **OR (95% CI)** | ***P* value** | **OR (95% CI)** | ***P* value** |
| TyG-BMI z-score | 1.44 (1.39, 1.50) | <0.001 | 1.43 (1.37, 1.50) | <0.001 | 1.32 (1.26, 1.39) | <0.001 |
| TyG-WHtR z-score | 1.68 (1.61, 1.75) | <0.001 | 1.48 (1.41, 1.55) | <0.001 | 1.33 (1.26, 1.40) | <0.001 |
| TyG-WC z-score | 1.48 (1.42, 1.54) | <0.001 | 1.45 (1.38, 1.51) | <0.001 | 1.30 (1.24, 1.37) | <0.001 |
| METS-IR z-score | 1.13 (1.09, 1.18) | <0.001 | 1.32 (1.26, 1.38) | <0.001 | 1.15 (1.10, 1.21) | <0.001 |
| HOMA-IR z-score | 1.16 (1.11, 1.21) | <0.001 | 1.12 (1.07, 1.17) | <0.001 | 1.09 (1.05, 1.14) | <0.001 |

Model 1: No covariates were adjusted.

Model 2: Adjusted for age, gender, and race.

Model 3: Adjusted for age, gender, race, education level, marital status, PIR, smoking status, alcohol consumption, diabetes, hypertension, CVD, stroke，Depression score，caffeine intake and carbohydrate intake.
